# Ecological analysis of health care utilisation for China's rural population: association with a rural county's socioeconomic characteristics

**DOI:** 10.1186/1471-2458-10-664

**Published:** 2010-11-02

**Authors:** Kam Ling Chau

**Affiliations:** 1 London Health Observatory, London, UK

## Abstract

**Background:**

The problem of accessibility and affordability of health care is reported to be a major social concern in modern China. It is pronounced in rural households which represent 60% of China's population. There are a few large scale studies which have been conducted into socioeconomic inequalities in health care utilisation for rural populations. Those studies that exist are mainly bivariate analyses. The aim of this study is to examine the relationship between socioeconomic characteristics and health service utilisation among rural counties, using aggregated data from a nationally representative dataset, within a multivariate regression analysis framework.

**Methods:**

Secondary data analysis was conducted on China's National Health Services Survey (NHSS) 2003. Aggregated data on health care utilisation, socioeconomic position, demographic characteristics and health status were used. The samples included 67 rural counties. Multivariate linear regression analyses were performed.

**Results:**

The results of the ecological multivariate analyses showed a positive relationship between private insurance coverage and the use of outpatient care (p-value < 0.05, standardised coefficient = 0.22). Annual income was positively correlated with annual medical expenditure (p-value < 0.01, standardised coefficient = 0.56). A rural county's area socioeconomic stratum, a composite measure frequently used in bivariate studies including the NHSS analysis report, could not explain any association with the use of health care.

**Conclusions:**

This study highlights that richer rural households with a greater ability to pay are more able to use health services in China. The findings suggest that the scope of medical insurance might be restrictive, or the protection provided might be limited, and the health care costs might still be too high. Additional efforts are required to ensure that poorer Chinese rural households are able to utilise health care according to their needs, regardless of their income levels or private insurance coverage. This would require targeted strategies to assist low income families and a broad spectrum of interventions to address the social determinants of health.

## Background

The last few decades have witnessed remarkable progress in economic growth and improvements in health status in China. China's GDP saw a 10-fold increase from 1988 to 2007. In 2006 the per capita income in China was US$ 2,025, which was nearly seven times the level of 1985 [1]. Going hand in hand with this are its achievements in overall population health, e.g. life expectancy at birth increased from 35 years before 1949 to 72 years in 2004 [2].

These national averages, however, mask differences in health status and differentials in access to and use of health care between different population groups. In the rural areas the under-5 mortality rate was 21.8 as opposed to 9.0 per 1,000 live births in urban areas in 2007 [3]; and urban residents took up a larger share of medical spending (78%) [4], despite the fact that over 60% of the population lives in rural areas.

Economic reforms and the associated liberalisation and privatisation of the health sector are a double-edged sword. Rapid economic growth has increased living standards greatly, lifting millions of people out of poverty [5]. At the same time, they have resulted in deepening social polarisation between income groups and across provinces. Problems of affordability of health services are particularly pronounced in the low-income population which lacks adequate income to purchase basic health care, let alone the impact of health care expenditure on the households' finances is high. Studies have reported that out-of-pocket health care payments coupled with the low level of medical insurance coverage have led to rural poverty in China [6,7].

Studies on the differentials in health services in China are very limited. Published studies have focused more on the disparities between urban and rural residents. Large scale or nation-wide health care utilisation studies involving rural populations are mainly bivariate analyses [8,9]. Bivariate analysis, however, ignores the presence of other possible confounding factors. There are a handful of multivariate regression studies which focus on a small number of rural counties, service for a particular health condition, or a particular health insurance scheme [10-18].

This study aims to examine the relationship between socioeconomic characteristics and health service utilisation among rural counties, using aggregated data from the National Health Service Survey (NHSS) within a multivariate analysis framework. Conducted every 5 years, the NHSS collects data on a large number of health status, health service utilisation, demographic and socioeconomic variables to construct a rich and nationally representative dataset to inform health policy formulation. At the time of writing, the latest available survey data were for 2003 and the 2008 survey was underway. This study remains relevant as it provides a baseline picture of within country health inequalities and allows monitoring of changes in health disparities over time when new NHSS data become available.

## Methods

The rural component of the dataset from the NHSS survey (2003) was used, which was the latest survey available at the time of this study. Data collected for individuals and individual households were published at a rural county/locality level. Individual-level data were not available.

### Data sampling strategy and procedures

The NHSS survey used a four-stage, stratified, random cluster-sampling design to identify a representative sample of the general Chinese population from its 32 administrative divisions (excluding Hong Kong and Macau) for interviews. Demographic, socioeconomic and health status indicators were used to classify 2,450 cities and rural counties into five strata (cities and four classes of rural counties - class I, II, III and IV), which were given different weights. The indicators for the classification of rural classes were: crude birth rate, crude death rate, infant mortality rate, literacy rate, percentage of the population with high-school education and beyond, percentage of the population over 65 years, percentage of the population under 15 years, percentage of the population engaged in the agricultural sector, percentage of the population engaged in the industrial sector, and percentage of the population engaged in the service sector. Class I rural counties have the best socioeconomic and health indicators whereas Class IV have the worst. Based on a sampling probability of 1:26 there were 67 rural counties and 28 cities chosen respectively according to their proportion to the total number of cites and counties (table 1) from across the five strata. The probability of each household being selected was about 1:4,912 in the survey. Households participating in the survey were randomly selected from the sample villages or residential committees that were used for the first and second NHSS. Overall, the 2003 survey encompassed a total of 143,991 rural residents representing 40,212 rural households.

**Table 1:** Sample size of the NHSS 2003

	Total number in China	Number of samples selected	Sampling probability
No. of cities/rural counties	2450	28 (cities) + 67 (counties) = 95	1:26

### Data collection and quality assurance

Local medical doctors, who underwent survey training, conducted household interviews through the use of a structured questionnaire. The past three surveys involved interviewing all the households' members - family and non-family members living together for over six months. Questions on marital status, education attainment and occupation were not applicable to interviewees under 15. The response rate of adult respondents was 77.8% for the 2003 survey.

Senior medical doctors at township level or above performed quality control. The quality assurance committee in each selected county chose 5% of the households sampled to repeat the questionnaire to make sure the results from the first and second interview were consistent. Survey supervisors performed the second visits and repeated data collection for 14 key indicators including: 2-week self-reported morbidity rate, number of inpatient visits over the preceding 52 weeks, distance to the nearest health care facilities, household's annual income and annual expenditure etc., to check for consistency of responses [19]. It is reported that the agreement rate was around 97% for the major indicators except the 2-week self-reported morbidity rate [20].

It is important to note that since the data collected by the NHSS were all self-reported, there was a possibility of recall bias. There were no other clinical measurements or verifications done to confirm the accuracy of these self-reports. The quality control measure mentioned above was done to minimise this bias.

### Ecological variables used

The unit of observations in the NHSS report is rural county.

#### Dependent variables

The number of outpatient visits and number of inpatient visits were chosen as the dependent variables for utilisation of outpatient and inpatient services respectively. These variables represent quantities of health service contacts. In China, self treatment and pharmacy visits are common practice. Therefore, per capita annual medical expenditure was used as a dependent variable. It represents the totality of monetary resource spent on improving the health of the individuals. These three dependent variables were largely complementary and mutually reinforcing.

#### Independent variables

Per capita annual income, medical insurance coverage and rural class were chosen as explanatory variables. The annual income represents the ability to pay for needed health care. Insurance is an important mechanism to ensure households have access to basic health services and provide financial protection against huge medical expenses. Rural class, an area socioeconomic stratum, is a common NHSS classification. Of particular interest are the differentials in health care utilisation between Class I and IV rural counties.

The percentage of households in a rural county having to travel more than 30 minutes to the nearest health facility was also chosen as it reflects the quality of the public transportation network and road infrastructure of the rural county in which people reside, and climate and geographical factors.

For the assessment of the respondents' state of health and illness, the number of self-reported restricted activity days and the number of doctor-diagnosed chronic conditions were taken as explanatory variables for the use of outpatient services. These two variables are considered to be comparatively more objective and reliable than the 2-week self-reported morbidity rate which was inconsistent when the survey was repeated [20]. As an indicator for the need of hospital care, the number of doctor referrals for hospitalisation was used.

Age could be a potential confounder. Three age strata were used: 0-14; 15-64 and over 65 years, as these three age strata are likely to differ in the need for and use of health care. Other demographic and socioeconomic variables - gender, martial status, educational attainment and occupation were not included because their data were in narrow ranges around their means (table 2), which makes regression analysis difficult. Furthermore, some of these variables were collected only from individuals over 15 years old. This could be problematic for use in statistical analyses where the selected dependent variables were for all individuals interviewed.

**Table 2:** Summary statistics of the rural counties

Variable†	Maximum	**3**^**rd **^**quartile**	Median	**1**^**st **^**quartile**	Minimum
Gender composition (%)					
- Male	54.9	52.0	51.1	50.1	46.7
- Female	53.3	50.0	48.9	48.1	45.1
					
Martial status (%)					
- Single	38.4	21.5	17.5	14.0	7.7
- Married	84.2	79.7	75.3	72.0	56.2
- Widowed	3.9	0.9	0.6	0.4	0.0
- Divorced	10.9	7.3	5.9	4.9	3.7
					
Educational attainment (%)					
- Illiterate & semi Illiterate	48.4	28.3	21.3	16.2	4.2
- Primary school (6 years)	50.1	35.8	30.4	26.5	19.3
- Junior high school (3 years)	55.0	44.2	37.2	29.1	5.4
- High school and/or post high school education	18.5	12.0	9.9	6.5	0.8
- University or above	1.6	0.4	0.2	0.1	0.0
					
Occupation (%)					
- Management	3.7	1.2	0.7	0.5	0.0
- Professional	3.6	1.4	0.8	0.5	0.1
- Office worker	12.9	4.2	2.4	1.1	0.2
- Factory worker	5.2	0.8	0.2	0.1	0.0
- Agriculture and farming labourer	96.0	89.5	84.0	78.6	57.1
- Unemployed & semi-unemployed	14.5	6.6	3.1	0.8	0.0
- Students & retired	14.8	8.5	7.4	5.6	2.7

^† ^Number of observations = 67 counties

Three regression models were used to test the relationship between outpatient visits, inpatient visits and annual medical expenditure, respectively, with the rural county socioeconomic and demographic characteristics. Table 3 details the variables used in the three models. All the variables are expressed in numerical values except for rural class.

**Table 3:** Ecological variables used in the analyses

Variable [short name]	Model 1	Model 2	Model 3
			
Number of Outpatient visits per 1000 population in the 2-week period prior to the survey [Outpatient visit]	*		
Number of Inpatient visits per 1000 population over the preceding 52 weeks [Inpatient visit]		*	
Per capita annual medical expenditure [annual medical expenditure]			*
			
Per capita annual income [Annual income]	*	*	*
Percentage of individuals in a rural county having government and social insurance [Government and social insurance]	*	*	*
Percentage of individuals in a rural county having private medical insurance [Private medical insurance]	*	*	*
Percentage of individuals in a rural county having no medical insurance cover and therefore to pay out of pocket for medical expenditure [No medical insurance cover]	*	*	*
Rural class IV	*	*	*
Rural class I	*	*	*
Percentage of households in a rural county having to travel more than 30 minutes to the nearest health facility [Time - Over 30 minutes to the nearest health facility]	*	*	*
Number of 2-week restricted activity days per 1000 population [Restricted activity days]	*		*
Number of doctor-diagnosed chronic conditions per 1000 population [Doctor-diagnosed chronic conditions]	*		*
Number of doctor referrals for hospitalisation per 1000 population [Doctor referrals for hospitalisation]		*	*
Age group - 0-4 and 5-14 years old	*	*	*
Age group - 15-24, 25-34,35-44, 45-54 and 55-64 years old	*	*	*
Age group - > 65+ years old	*	*	*

### Statistical methods

There is no previous empirical research using the NHSS dataset on the concerned topic that provides causal models to explain healthcare utilization. In this study, linear regression was used to explore whether the simplest linear relationship exists between the dependent variable and independent variables from statistical notions.

Univariate ecological analyses were carried out to inform which explanatory variables should be included in multivariate analyses. A cut-off point of p-value < 0.05, which is commonly regarded as statistically significant, was used to select which independent variables should be fitted into the multivariate regression analyses.

Multiple linear regression was then conducted. The assumptions for multiple linear regression were checked: by producing graphical plots of regression standardised residuals against regression standardised predicted values to ensure that there was a random cloud of points, and by producing histograms of residuals to ensure that the regression standardised residuals were normally distributed with a mean of 0 and within the range of +2 and -2. Multicollinearity of the explanatory variables was checked against Tolerance/VIF values to make sure that there were no near perfect combinations among them. The values of Tolerance must be > 0.1 and those of VIF must be < 10 to ensure the assumptions for multiple linear regression were met. As a precaution, autocorrelation (correlation among the data of the dependent variable) was checked by running Durbin-Watson tests to ensure the Durbin-Watson values were around the range of 1.5 - 2.5. The results indicating there were no correlations among data of dependent variables. All statistical analysis procedures were performed using SPSS version 15.0.

The three models of multiple linear regression were compared by means of adjusted R squared (adjusted R^2^) value to measure the overall goodness of fit. For each model both unstandardised and standardised coefficients and p-values for independent variables were reviewed. Typically, standardised coefficients less than 0.10 (in absolute value) are 'trivial' or are non-significant if p-value > 0.05.

## Results

### Descriptive analysis

Table 4 provides the characteristics of the variables in this study. The share of medical spending to total income was 10.5% on average for the total rural population, ranging from 4.5% in a high-income rural county to 21.8% in a low-income one. There were 12 rural counties with average annual savings less than 100 Yuan (US$14.9) which left these households no provision for sudden incidence of ill-health. It is also alarming to note that majority of rural counties had very low or almost no medical insurance of any type at all. In other words, the proportion of the population in these rural counties having to pay for their own health care was very high.

**Table 4:** Characteristics of the study variables

Variable†	Maximum	**3**^**rd **^**quartile**	Median	**1**^**st **^**quartile**	Minimum
Outpatient visits	348.9	177.7	130.5	86.8	34.5
Inpatient visits	94.8	41.4	29.7	24.8	8.8
Per capita annual medical expenditure (Yuan)	603.0	261.0	216.0	174.0	79.0
Per capita annual income (Yuan)	7424.0	2595.0	2122.0	1542.0	951.0
Government & social insurance (%)	95.5	8.6	3.2	1.8	0.1
Private medical insurance (%)	25.2	11.8	6.4	3.5	0.2
No medical insurance cover (%)	98.1	93.1	85.8	75.4	4.3
Rural class IV‡					
Rural class I‡					
Time - over 30 mins to the nearest health facility (%)	36.2	9.3	2.7	0.2	0.0
Number of restricted activity days	384.0	220.0	162.0	114.0	51.0
Number of doctor-diagnosed chronic conditions	236.5	126.1	104.3	84.5	28.9
Number of doctor referrals for hospitalisation	139.2	55.5	41.4	35.8	13.9
Age 0-14 (%)	35.5	25.2	22.2	18.9	11.5
Age 15-64 (%)	79.4	72.0	70.4	66.9	57.7
Age > 65+ (%)	17.4	9.7	7.7	6.1	2.9

^† ^Number of observations = 67 counties.

^‡ ^Summary statistics are not applicable for this variable

### Multivariate ecological analyses

From the results of univariate analyses, the independent variables chosen to be included in the multivariate ecological analyses were:-

• For model 1 (outpatient service use) - private medical coverage, restricted activity days, doctor-diagnosed chronic conditions, and age group 15-64

• For model 2 (inpatient service use) - doctor referrals for hospitalisation

• For model 3 (medical expenditure) - annual income, private medical coverage, rural class I, rural class IV, percentage of households having to travel more than 30 minutes to the nearest health facility, doctor-diagnosed chronic conditions, doctor referrals for hospitalisation, age group 0-14 and age group over 65

The explanatory variables in model 1 explained 33.9% of variation seen in the use of outpatient services (table 5). Private medical insurance and doctor-diagnosed chronic conditions were statistically significantly associated with the use of outpatient services. People aged 15-64 had an inverse association with outpatient service use.

**Table 5:** Coefficients and p-values of multivariate regression analysis of model 1

Variable	Coefficient	Standard Error	Standardised Coefficient	p-value
Constant	508.153	133.801		0.000
				
Private medical insurance	227.961	104.599	0.221	0.033
Restricted activity days	0.214	0.111	0.212	0.058
Doctor-diagnosed chronic conditions	0.446	0.189	0.257	0.022
Age 15-64	-6.765	1.871	-0.372	0.001

Dependent variable: 2-week outpatient visits per 1000 population

(Adjusted R^2 ^= 0.339, Durbin-Watson = 1.461, F = 9.447, Sig = 0.000)

Doctor referrals for hospitalisation was the only variable and was statistically significant in model 2 (table 6).

**Table 6:** Coefficients and p-values of univariate regression analysis of model 2

Variable	Coefficient	Standard Error	Standardised coefficient	p-value
Constant	8.420	2.565		0.002
				
Doctor referrals for hospitalisation	0.522	0.048	0.806	0.000

Dependent variable: inpatient visits per 1000 population

(Adjusted R^2 ^= 0.644, Durbin Watson = 1.978, F = 120.531, Sig = 0.000)

Model 3 explained 62.5% of variation. From the standardised coefficients, explanatory variables with the largest relative association with per capita annual medical expenditure were, in descending order, annual income, doctor-diagnosed chronic conditions and doctor referrals for hospitalisation (table 7). With other chosen variables held constant, annual income had a greater effect on annual medical expenditure (standardised coefficient = 0.56, p-value = 0.000). Rural class variables could not explain the difference in medical expenditure among rural counties. Health status, which was measured by the number of doctor-diagnosed chronic conditions and the number of doctor referrals for hospitalisation, was positively associated with medical expenditure.

**Table 7:** Coefficients and p-values of multivariate regression analysis of model 3

Variable	Coefficient	Standard Error	Standardised Coefficient	p-value
Constant	-19.689	80.851		0.808
				
Annual income	0.055	0.010	0.560	0.000
Private medical insurance	-39.840	132.916	-0.024	0.765
Rural class IV	16.233	36.544	0.050	0.659
Rural class I	-2.885	23.552	-0.012	0.903
Time taken over 30 mins to the nearest health facility	-212.677	123.693	-0.192	0.091
Doctor-diagnosed chronic conditions	0.572	0.251	0.209	0.027
Doctor referrals for hospitalisation	0.823	0.393	0.179	0.041
Age 0-14	0.215	2.553	0.009	0.933
Age 65+ years old	5.115	3.449	0.142	0.144

Dependent variable: per capita annual medical expenditure

(Adjusted R^2 ^= 0.625, Durbin-Watson = 1.886, F = 13.206, Sig = 0.000)

## Discussion

The regression analyses reveal a positive relationship between worsening health status and health care utilisation, suggesting that those who are ill have access to some form of health care - typically professional treatment or self medication. Private medical insurance cover was also positively associated with the use of outpatient services (model 1) but not the other forms of health care. Government and social insurance coverage did not have any impact in any of the 3 models. It suggests the scope of insurance coverage, regardless of type, might be restrictive or the protection provided might be very limited. The association demonstrated between health care utilisation and income levels (model 3) was in line with findings from previous multivariate studies (Zhang T et al and Wang H et al., etc.). A higher level of annual income was independently associated with annual medical expenditure, suggesting that richer households with a greater ability to pay are more able to use health service (model 3).

A rural county's area socioeconomic stratum (rural class), a composite measure, could not explain any association with the use of health care when controlling for other relevant factors of interest. This is contrary to the reported variations in the use of health care by rural class published in other bivariate studies including NHSS analysis report.

This study suggests that, in a health system like China's, where out-of-pocket is the dominant form of payments for health care, household income is one of the deciding factors for the use of health care. Expanding the government and social health insurance to eventually cover the entire rural population could lower the financial barriers to equitable access of care. The Chinese government has been attempting to achieve this through the new (rural) Cooperative Medical Scheme (NCMS). The new scheme operates at county level with government subsidy to match contributions made by households. At the time the 2003 survey was conducted, the NCMS was in its early pilot stage and only implemented in a few selected counties. As of 2008, the scheme has been rolled out to 2,729 rural counties covering a population of 810 million. The percentage enrolled in the scheme was 91.5% [4].

It is however not only the presence of insurance coverage that matters but also the level of the protection and the scope of the coverage. There is a need to scale up the contributions to the NCMS jointly from households and the government to a level that matches current per capita medical expenditure. The NCMS enrolees are paying per capita 96.3 Yuan (US$ 14.3) premium per year. Hospital care, however, is far more expensive in China - 5,897 Yuan (US$ 879) per one hospital admission as compared to 155 Yuan (US$ 23) per one outpatient visit [21]. Some studies have already pointed out that the NCMS offers very limited protection in terms of the type of care and amount of health care costs covered and does not necessarily reduce the burden of out-of-pocket expenditure on households [22-24]. An increase in the amount of contributions to the scheme by the government and enrolees could provide a reasonably large and more heterogeneous risk pool. It could also lower the average risk of the scheme and extend the coverage of the scheme to those areas such as hospital care or co-payment for medication for serious chronic conditions.

The autonomy of a rural county to finance, design and manage the NCMS could also adversely affect people, especially for those living in cash strapped counties. A county's ability to contribute to NCMS depends very much on their per capita income when fiscal transfers between provinces are very limited [25]. A mechanism is therefore required to pool funds and equalise resources across local governments to ensure that the poorer rural counties have the financial subsidy they need to put in place a viable insurance scheme. Different NCMS programme designs could influence access to and utilisation of health care [17,26]. However for a vast country like China with different levels of socioeconomic development across rural counties, it is difficult, if not unfeasible, to have a one-size-fits-all health insurance model. Future analysis using the 2008 survey data to assess the full impact of the NCMS, alongside studies on what worked and what did not work, and the sharing of best practice could help improve the health care financing model for the rural populations.

On the other side of the equation, the Chinese expect to have medicines prescribed at each doctor visit and have the false expectations that the more expensive drug work better. When unnecessary prescriptions are made or expensive drugs are often used instead of cheaper alternatives by doctor or self prescription, it is more likely to leave a coverage gap even for the insured, and create substantial out-of-pocket financial burden (table 4). Health education is therefore required to change the public perception about the rational use of medicines and that the efficacy of a drug does not necessarily come with a high price tag.

Given widespread dissatisfaction and frustration with the health system, the Chinese Government has recently unveiled a major reform plan with the aim of providing universal health care for all by 2020. In an effort to tackle health inequalities, investment priority will be given to the most deprived areas of the country like central and western China. It is imperative to ensure that the reform will benefit the most disadvantaged subgroups of the population. Government expenditure as a proportion of the total health expenditure has increased from 36.2% in 2003 to 45.3% in 2007 [27]. However, previous experience shows that government spending on health in China tends to benefit the rich disproportionately [28,29]. Even with the full roll out of the NCMS, it does not necessarily help narrow the health inequalities gap. A targeted strategy that focuses on removing the barriers to health care and addressing the specific needs of the poorest segment of society is required to overcome the 'inverse care law'. Success stories from other low-income countries such as some African and Southeast Asian countries [30-33] could provide insights into a more equitable financing and provision of health care in China. To this end, the use of meaningful health intelligence underpinned by systematic and robust data collection is integral to the planning and prioritisation of health resources for the target groups.

Last but not the least, improving access to health services requires tackling the wider determinants of health such as education, housing and poverty [34]. This will require joint efforts of Ministries across the Government and collaboration with the private and third sectors on improving the living conditions and life chances of the rural populations.

Ecological analysis can provide a wealth of useful observations. However, there are limitations. This study is subject to ecological fallacy. The magnitude of correlations found at the group level in this study should not be assumed to be consistent with the observations at individual level because of confounding factors or modification of effect. There is also a possibility that the reported associations at the group level may be confounded by factors that have not been gathered in the NHSS or included in the hypotheses for testing. The latter is owing to the fact that some variables such as the number of households in poverty do not have clear and concise definitions in the NHSS report which render them unsuitable for use in this study.

Furthermore, different levels of aggregation in the same ecological study may not give identical results [35]. The selection of an appropriate unit of aggregation needs to be carefully considered for an ecological analysis when individual-level data are available for aggregation at one's disposal. In this study, the choice of appropriate geographic unit of analysis was restricted by the availability of data. Rural counties were used as they are the smallest unit of geographical data available in the NHSS report.

While ecological studies allows a quick and inexpensive way to assess the association between outcomes and factors of interest, the inability of ecological data to characterize potential between-and within-group confounders often make it difficult to draw definitive conclusions. There are several strategies [36-39] that could be used to address potential ecological biases. These include supplementing ecological studies with individual-level information, the use of simulated data and statistical procedures like Bayesian methods. These statistical procedures however are fairly complex and not regularly used by public health practitioners. Further studies using NHSS individual record data when made available for international researchers are required to examine and verify the association reported in this study.

The relevance of ecological variables, however, should not be ignored as health policy is usually implemented at community, regional or national level, rarely at individual level. Ecological variables can be very useful for the study of variations in health and health care utilisation between geographical areas [40,41].

This analysis helps lay the foundation for further research by establishing a baseline picture against which measurement of change in the health inequalities can be made with 2008 NHSS data and beyond. Regular monitoring and progress tracking toward the reduction of health inequalities could help inform targeted policy interventions. Trend analysis would be particularly useful as the impact of major health interventions will take years to show, individual subgroups may achieve different levels of improvement, and new inequalities may arise over time.

## Conclusions

To summarise, the study results suggest socioeconomic inequalities exist in access to and the use of health care. The use of health care was associated with income level, a factor contributing to the reported inequalities in health care utilisation. Mean level of private medical insurance coverage was only found to be associated with the use of outpatient care. Government insurance did not have impact on any types of health care. One possible explanation is that the scope of insurance coverage and level of protection may be very limited. While ecological analysis cannot identify causal relationships, income levels and medical insurance coverage factors may have health policy implications. Social and economic policies that affect income distribution may have important consequences for the use of health care.

The expressed concern - 'getting medical service is difficult, getting medical service is expensive' [42] - is not only about Chinese dissatisfaction with affordability and accessibility of health care, but also the quality of health care. While achieving improved health status is one of the key targets any health care system should strive to achieve, improving service users' confidence and satisfaction with the system is equally important. The latter is an important area for future research.

## Competing interests

The author declares that they have no competing interests.

## Authors' contributions

KL Chau authored this manuscript.

This manuscript does not represent the view of London Health Observatory, Commissioning Support for London or its host organisation NHS Camden.

## Pre-publication history

The pre-publication history for this paper can be accessed here:

http://www.biomedcentral.com/1471-2458/10/664/prepub
